# Straw from Different Crop Species Recruits Different Communities of Lignocellulose-Degrading Microorganisms in Black Soil

**DOI:** 10.3390/microorganisms12050938

**Published:** 2024-05-05

**Authors:** Chunling Chang, Yue Guo, Kuanqiang Tang, Yunlong Hu, Weihui Xu, Wenjing Chen, Neil McLaughlin, Zhigang Wang

**Affiliations:** 1 College of Life Science and Agroforestry, Qiqihar University, Qiqihar 161006, China; changchunling0709@163.com (C.C.); 15245222062@163.com (Y.G.); tangkuanqiang@126.com (K.T.); hu13836256517@163.com (Y.H.); xwh800206@163.com (W.X.); 03805@qqhru.edu.cn (W.C.); 2 Heilongjiang Provincial Technology Innovation Center of Agromicrobial Preparation Industrialization, Qiqihar 161006, China; 3 Heilongjiang Provincial Collaborative Innovation Center of Agrobiological Preparation Industrialization, Qiqihar 161006, China; 4 Ottawa Research and Development Centre, Agriculture and Agri-Food Canada, Ottawa, ON K1A 0C6, Canada; neil.mclaughlin@sympatico.ca

**Keywords:** black soil, crop straw, KEGG pathways, lignocellulose-degrading microbes, microbial community structure, synthetic microbial communities

## Abstract

The biological degradation of plant residues in the soil or on the soil surface is an integral part of the natural life cycle of annual plants and does not have adverse effects on the environment. Crop straw is characterized by a complex structure and exhibits stability and resistance to rapid microbial decomposition. In this study, we conducted a microcosm experiment to investigate the dynamic succession of the soil microbial community and the functional characteristics associated with lignocellulose-degrading pathways. Additionally, we aimed to identify lignocellulose-degrading microorganisms from the straw of three crop species prevalent in Northeast China: soybean (*Glycine max* Merr.), rice (*Oryza sativa* L.), and maize (*Zea mays* L.). Our findings revealed that both the type of straw and the degradation time influenced the bacterial and fungal community structure and composition. Metagenome sequencing results demonstrated that during degradation, different straw types assembled carbohydrate-active enzymes (CAZymes) and KEGG pathways in distinct manners, contributing to lignocellulose and hemicellulose degradation. Furthermore, isolation of lignocellulose-degrading microbes yielded 59 bacterial and 14 fungal strains contributing to straw degradation, with fungi generally exhibiting superior lignocellulose-degrading enzyme production compared to bacteria. Experiments were conducted to assess the potential synergistic effects of synthetic microbial communities (SynComs) comprising both fungi and bacteria. These SynComs resulted in a straw weight loss of 42% at 15 days post-inoculation, representing a 22% increase compared to conditions without any SynComs. In summary, our study provides novel ecological insights into crop straw degradation by microbes.

## 1. Introduction

The Northeast China black soil region stands as one of the four primary black soil regions worldwide, contributing to 20% of the country’s crop production and serving as the largest commercial crop production base in China [1]. The main food crops in this region are soybean (*Glycine max* Merr.), rice (*Oryza sativa* L.), and maize (*Zea mays* L.). In recent years, there has been a significant surge in the annual generation of crop straw, posing challenges for field operations to cultivate crops for the following year [2]. Traditional straw disposal methods, such as removal, burning, and burial in soil, have their drawbacks. Removal requires additional labor, while widespread burning contributes to severe air pollution [3], a concern that led to its prohibition in Northeast China. Both removal and burning are inefficient and wasteful, as crop straw is a rich source of carbon resources, primarily lignocellulose. When incorporated into the soil and allowed to decompose, this organic material enhances soil health by releasing nutrients for crop growth and adding soil organic carbon [4]. The natural decomposition of buried straw in the soil is slow, and intact straw brought to the surface during tillage operations for subsequent crops can obstruct field machinery. Therefore, finding a pollution-free solution for straw disposal is crucial for promoting green agriculture and improving resource utilization rates.

In recent research, a novel technique for crop straw biodegradation employing specific microbial communities has been investigated. This method is environmentally friendly, requires minimal energy input, is cost-effective, and operates under mild conditions [5]. Microorganisms imparting biodegradation encompass bacteria, fungi, and actinomycetes [6]. Nevertheless, studies focusing on lignocellulose-degrading microorganisms indicate that fungi are sensitive to surrounding environmental conditions and are unsuitable for large-scale industrial production [7,8]. Conversely, bacteria exhibit a lower lignin degradation capacity compared to fungi [9]. Therefore, it is crucial to explore potential decomposer microorganisms to accurately predict their performance in degrading lignin from crop straw.

Specific microbes capable of degrading lignin establish on the surface of straw and act as decomposing agents [10]. These microbes produce cellulose-degrading enzymes that break down long-chain cellulose polymers into smaller molecular weight or monomeric compounds [11]. Crop straw exhibits a complex structure, including covalent bonds with hemicellulose and proteins embedded within cellulose, thereby enhancing its stability and resistance to degradation by single microbial taxa [12,13]. Rather than employing the traditional “one-microbe-at-a-time” approach [14], an alternative strategy involves creating synthetic microbial communities (SynComs). In these communities, the synergy of different taxa may enhance the degradation rate of straw from various crop species [15].

In this study, we employed a comprehensive methodology to investigate the degradation times of various straw types by indigenous black soil microorganisms. Additionally, we isolated different types of lignocellulose-degrading microorganisms. The specific objectives were as follows: (1) quantify the impact of three common crop straw types and their decomposition times on the soil microbiome structure and composition through amplicon sequencing, (2) identify changes in straw degradation by analyzing prominent biomarker microorganisms in KEGG pathways and enzymatic pathways through metagenome sequencing, (3) isolate lignocellulose-degrading microorganisms and construct SynComs using artificially synthesized microbiomes, and (4) assess the functionality of synthetic microbial communities during crop straw degradation.

## 2. Material and Methods

### 2.1. Soil Preparation and Straw Characterization

The black soil (Mollisols in the US taxonomy system) was gathered from chernozem in northeastern China in Keshan County, Qiqihar City, Heilongjiang Province (48°21′ N, 126°03′ E) [16]. Surface layer (0–20 cm depth) soil samples were obtained from six sites within a 50 m area and sifted through a 2-mm screen to remove stones and organic materials to create a composite sample. Subsequently, the soil samples were sealed in airtight plastic bags, which were placed on ice for transport to the laboratory. In the laboratory, all soil samples were subsequently homogenized and subdivided; for one part, soils were stored at −80 °C for subsequent analysis of 16S rRNA and ITS sequencing. For the other part, soils were air-dried at room temperature for subsequent soil physicochemical property measurements. Soil properties had the following values: 7.77 pH (1:2.5, *w*/*v*), 13.8 μs/cm electric conductivity (1:2.5, *w*/*v*), 2.31 g/kg total nitrogen, 6.02% soil organic matter, 6.85 mg/kg available phosphorus, and 159 mg/kg available potassium.

Crop straws were collected from a local farm in Qiqihar, China (47°35′ N, 123°92′ E); the chemical properties of the soybean, rice, and maize straw were 41.2%, 37.1%, and 40.6% total carbon, respectively, and 0.94%, 0.69%, and 0.72% total nitrogen, respectively. Air-dried crop straws were cut into 3–4 cm pieces [17] and then evenly mixed with black soil at a 1:10 (wt:wt) ratio in pots. Soil moisture was adjusted to 60% of field capacity by adding water, and this level was maintained by periodic weighing and adding water as needed. Pots were covered to minimize evaporation and placed in an incubator at 30 °C for 180 days. Soil samples were collected from the surface of the straw using a brush on days 0, 90, 120, and 180 [18]. Each group comprised five biological replicates. Fresh soil samples from each sampling date were stored in a −80 °C freezer for subsequent analysis for microbial community structure and diversity.

### 2.2. DNA Extraction and Sequencing Analyses

The genomic DNA extraction from soil samples was conducted using the E.Z.N.A.^®^ soil DNA Kit according to the manufacturer’s guidelines (Omega Biotek, Norcross, GA, USA). The hypervariable V3–V4 regions of the bacterial 16S rRNA gene were amplified with primer pairs 338F/806R [19]. Additionally, the ITS genes were amplified using primer pairs ITS1F/ITS2R on an ABI GeneAmp^®^ 9700 PCR thermocycler (Applied Biosystems, San Diego, CA, USA). Subsequently, PCR segments were obtained from a 2% agarose gel, purified with an AxyPrep DNA Gel Extraction Kit (Axygen Biosciences, Union City, CA, USA), and quantified using a Quantus Fluorometer (Promega, Madison, WI, USA).

Paired-end amplicon sequencing (Appendix A) was performed on an Illumina MiSeq PE300 platform (Illumina, San Diego, CA, USA). For downstream bioinformatics analysis, raw sequences were demultiplexed based on barcodes and adaptors, and primer sequences were removed using Quantitative Insights into Microbial Ecology 2 (QIIME2) [20]. DADA2 was employed for denoising by filtering out sequences with an abundance of less than five. The final amplicon sequence variants (ASVs) with 100% similarity were obtained [21]. Taxonomy was assigned by the q2-feature-classifier [22] with the classify-sklearn method against the UNITE reference database [23]. Purified amplicons were equimolarly pooled and subjected to paired-end sequencing on an Illumina MiSeq PE300 platform (Illumina, San Diego, CA, USA) following the instructions of Majorbio Bio-Pharm Technology Co., Ltd. (Shanghai, China) [24].

Metagenomic sequencing (Appendix A) was conducted on an Illumina NovaSeq (Illumina Inc., San Diego, CA, USA) at Majorbio Bio-Pharm Technology Co., Ltd. (Shanghai, China) using a NovaSeq 6000 S4 Reagent Kit v1.5 (300 cycles) following the manufacturer’s instructions. Briefly, paired-end Illumina reads were trimmed of adaptors, and low-quality reads (length < 50 bp, with a quality value < 20, or having N bases) were removed using fastp (version 0.20.0). The metagenomic data were assembled using MEGAHIT (version 1.1.2), employing succinct de Bruijn graphs. Contigs with a length ≥ 300 bp were selected as the final assembly result, followed by gene prediction and annotation.

### 2.3. Isolation and Characterization of Lignocellulose-Degrading Microorganisms

The straw lignocellulose-degrading bacteria were isolated from soil samples collected at various sampling dates using the serial dilution plate method [25]. To initiate the isolation process, 1 g of soil obtained from the straw surface was thoroughly mixed and added to 9 mL of sterile water in a shaker set at 30 °C and 120 rpm for 20 min. Subsequently, the soil suspension was serially diluted and inoculated onto a straw agar medium consisting of 20 g/L of commercial straw powder (<1 mm) and 1 g/L of urea and adjusted to a pH of 7.0. Following a 7-day incubation period at 30 °C, single colonies were selected and cultured on the same medium to achieve purification. Genomic DNA extraction was performed employing a bacterial genomic DNA extraction kit (Tiangen Biochemical Technology Co. Ltd., Beijing, China). Bacteria and fungi were identified using the primers 27F/1492R and ITS1/ITS4, respectively. The obtained 16S rRNA and ITS gene sequences were compared using the Ezbiocloud and NCBI databases. After the removal of potential clonal duplicates, a total of 59 bacterial and 14 fungal strains were successfully isolated, and their glycerol stocks were prepared and stored at −80 °C.

### 2.4. Lignocellulose-Degrading Enzyme Activity

The strains were initially pre-cultured in a liquid beef paste peptone medium for 24 h and subsequently inoculated into a liquid minimal medium adjusted to a pH of 7.0 [26]. Straw was employed as the exclusive carbon source in the medium, and the cultures were incubated on a shaker at 30 °C and 150 rpm for 7 days. Following this incubation period, the broth underwent centrifugation at 4 °C and 12,000 rpm for 10 min, after which the supernatant was subjected to a secondary centrifugation to separate cellulase, xylanase, and laccase. Enzyme activity was quantified using enzyme activity kits procured from Grace Biotechnology (Suzhou Grace Biotechnology Co., Ltd., Suzhou, China) following the manufacturer’s instructions. Straw-degrading SynComs were assembled by combining strains in equimolar ratios guided by their metagenomic and enzyme-producing capabilities. The remaining straw in the liquid medium was harvested, dried to a constant weight, and the straw weight loss rate was computed.

### 2.5. Statistical Analysis

Statistical analyses were conducted using R version 4.0.3 [27]. Alpha diversity was computed employing the Shannon and Simpson indices via the *OTU.diversity* function in the RAM package [28]. Microbial β-diversity was evaluated using non-metric multidimensional scaling (NMDS) based on Euclidean and Bray–Curtis dissimilarity metrics for normalized ASV data of bacteria and fungi, respectively [29]. To examine the impact of straw type and degradation times on community dissimilarity, permutational multivariate analysis of variance (PERMANOVA) was performed using the “vegan” package. Kyoto Encyclopedia of Genes and Genomes (KEGG) annotation profiles corresponding to the genes were obtained from the KEGG database. Reporter scores, based on Z-scores, were utilized to statistically assess all KEGG orthologs (KOs) involved in a pathway. Reporter scores exceeding 2.58 or falling below −2.58 (99% confidence) were set as the detection threshold. Annotation profiles of carbohydrate-active enzyme genes were acquired by comparing them with the Carbohydrate-Active enZymes database (CAZyme) [30]. Statistical evaluation of significantly different abundances of taxonomic metagenomes was conducted using the linear discriminant analysis (LDA) effect size with LEfSe analysis [31]. The Wilcoxon test was employed for comparing two groups, while for more than two groups, a one-way analysis of variance (ANOVA) followed by Tukey’s honestly significant difference (HSD) test was performed to determine statistical significance among treatments.

## 3. Results

### 3.1. Effects of Straw Degradation on Soil Microbial Community Structure and Composition

**Figure 1:**
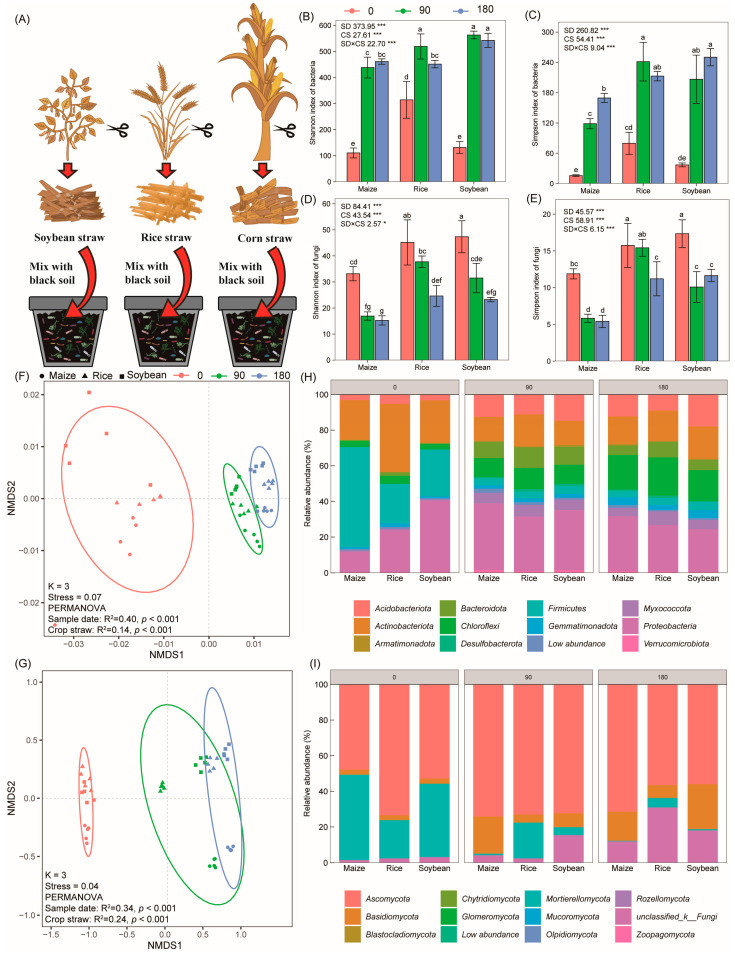
Changes in microbial community diversity and structure in the soil on the surface of crop straw. (**A**) Diagram illustrating the method of preparing straw for the degradation experiment. (**B**) Shannon index at the bacterial level. (**C**) Simpson index at the bacterial level. (**D**) Shannon index at the fungal level. (**E**) Simpson index at the fungal level. (**F**) NMDS with Euclidean distance showing bacteria on degradation days 0, 90, and 180. (**G**) NMDS with Bray–Curtis distance analyzing fungi on degradation days 0, 90, and 180. Ellipses cover 90% of the data for each treatment. (**H**) Phylum-level distribution of the bacterial community. (**I**) Phylum-level distribution of the fungal community. Effects of time on straw degradation (SDs), different crop straws (CS), and their interaction are shown as the F value with the test of significance (* *p* < 0.05; *** *p* < 0.001) of the two-way ANOVA. Error bars represent standard errors with five biological replicates. Different lowercase letters indicate significant differences (*p* < 0.05).

Straw degradation significantly increased bacterial α-diversity and decreased fungal α-diversity as indicated by the Shannon and Simpson indices. A significant two-way interaction between crop straw type and degradation time was observed (Figure 1B–E). Notably, maize straw exhibited the lowest bacterial and highest fungal α-diversity, indicating that maize degradation recruited less bacterial species and more fungal species than soybean or rice straw. Consistent with these findings, the non-metric multidimensional scaling analysis (NMDS) revealed significant alterations in bacterial (PERMANOVA, *p* < 0.001) and fungal (PERMANOVA, *p* < 0.001) phylogenetic β-diversities with respect to straw types and degradation time (Figure 1F,G). Proteobacteria, Actinobacteria, and Firmicutes were the main bacterial phyla, while Ascomycota, Mortierellomycota, and *Basidiomycota* were the dominant fungal phyla across the different crop straw types (Figure 1H,I).

### 3.2. Changes in Functional Characteristics in Degradation of Different Straw Types

**Figure 2:**
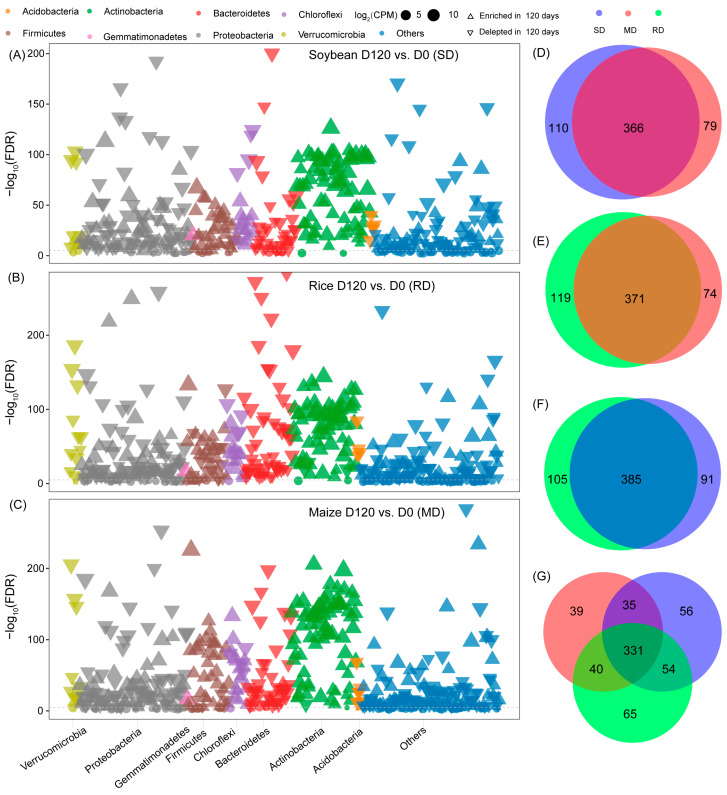
Taxonomic characteristics of different bacteria in the degradation of three crop straw types. (**A**) Manhattan plot showing enriched families in soybean for 120 vs. 0 days of degradation. (**B**) Manhattan plot showing enriched families in rice for 120 vs. 0 days of degradation. (**C**) Manhattan plot showing enriched families in maize for 120 vs. 0 days of degradation. (**D**) Overlapping enriched families in soybean and maize for 120 vs. 0 days of degradation. (**E**) Overlapping enriched families in rice and maize for 120 vs. 0 days of degradation. (**F**) Overlapping enriched families in soybean and rice for 120 vs. 0 days of degradation. (**G**) Overlapping enriched families among soybean, rice, and maize for 120 vs. 0 days of degradation. Each triangle represents the bacterial phylum (log_2_(fold change) > 1, FDR adjusted *p* < 0.05).

To obtain a more comprehensive perspective, we selected 18 DNA samples from the three crop straw types at both 0 and 120 days of straw degradation and conducted metagenomic sequencing to unveil the functional characteristics. Initially, we analyzed the enriched families based on their taxonomy using Manhattan plots (Figure 2A–C). Among the three types of crop straw, the species that exhibited greatest enrichment after 120 days of degradation belonged to a diverse array of bacterial phyla, including Acidobacteria, Proteobacteria, Actinobacteria, Bacteroidetes, and Firmicutes (Figure 2A–C). Additionally, we observed a significant overlap among soybean, rice, and maize-enriched families after 120 days of degradation; specifically, 331 families were enriched across the three straw types (Figure 2D–G).

**Figure 3:**
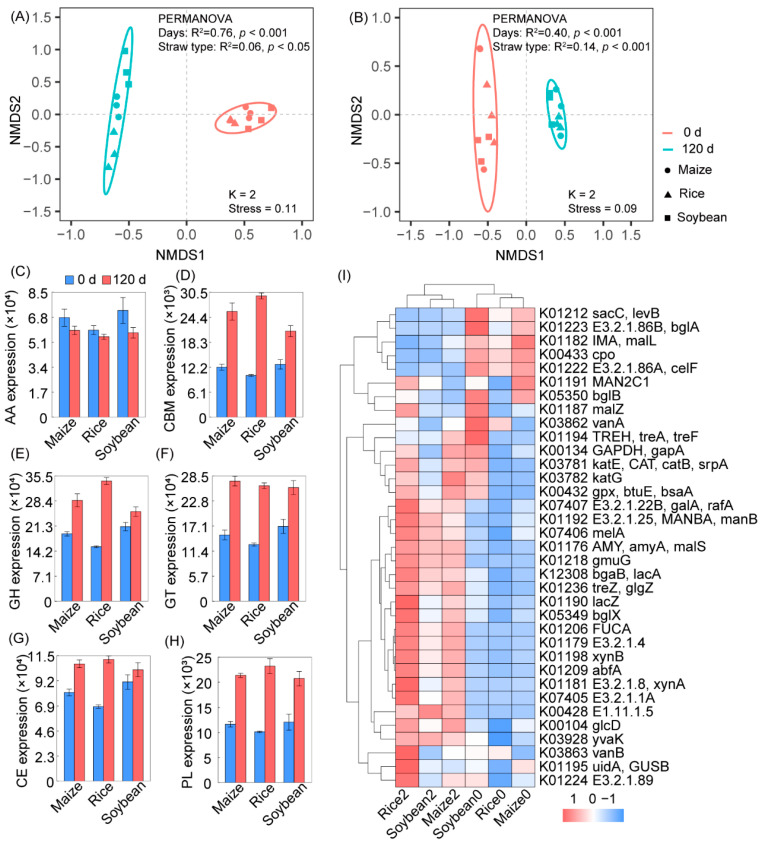
Functional characteristics of different bacteria in the crop straw degradation process in the KEGG and CAZymes database. (**A**) NMDS with Bray–Curtis distance analysis of KOs in three straw types after 120 days of degradation. (**B**) NMDS with Bray–Curtis distance analysis of CAZymes in three straw types after 120 days of degradation. Ellipses cover 90% of the data for each treatment. (**C**–**H**) Expression of six CAZymes. (**I**) The difference in KEGG pathway of straw degradation after 120 days. Error bars represent standard errors with three biological replicates.

After establishing taxonomic differences, we conducted an analysis of functional compositions, specifically the NMDS ordination of KOs and CAZymes. The results demonstrated a significant variation in the soil microbiome community after 120 days of straw degradation time (PERMANOVA, *p* < 0.001; see Figure 3A,B). To further elucidate the specialized and distinct functional profiles among the various crop straw types at the two degradation time points, we searched the carbohydrate-active enzyme (CAZyme) database associated with cellulose, hemicellulose, lignin, and cellulose oligosaccharide degradation. Our findings indicated a substantial increase in the activities of polysaccharide lyases (PLs), glycosyl transferases (GTs), glycoside hydrolases (GHs), and carbohydrate esterases (CEs) after 120 days of degradation (see Figure 3C–H). Additionally, we observed significant upregulation of the cellobiose hydrolysis gene (*bglX*) and hemicellulose degradation genes (*lacZ*, *xynA*, and *abfA*) following crop straw degradation. Moreover, the crop straw exhibited enrichment in genes with KEGG Orthologs (KOs) related to lignin and hemicellulose degradation, with notable increases in K07406, K01218, K01179, and K00428 (Figure 3I).

To assess the similarities and differences in KOs across various crop straw types at both 0 and 120 days of degradation, reporter scores based on KEGG pathways were analyzed. The analysis revealed that after 120 days of degradation, several pathways associated with the biosynthesis of amino acids, biosynthesis of nucleotide sugars, amino sugar and nucleotide sugar metabolism, bacterial secretion system, flagella assembly, o-antigen nucleotide sugar biosynthesis, 2-oxocarboxylic acid metabolism, biosynthesis of cofactors, lysine biosynthesis, carbon metabolism, carotenoid biosynthesis, lipopolysaccharide biosynthesis, sulfur metabolism, and biotin metabolism were significantly enriched. In contrast, on the initial sampling date (day 0), only seven KEGG pathways showed enrichment: arabinogalactan biosynthesis, phenylalanine metabolism, retrograde endocannabinoid signaling, biosynthesis of ansamycins, the phosphotransferase system, furfural degradation, and degradation of aromatic compounds (Supplementary Figure S3).

### 3.3. Isolation of Lignocellulose-Degrading Microorganism Strains and Determination of Enzyme Production Capacity

**Figure 4:**
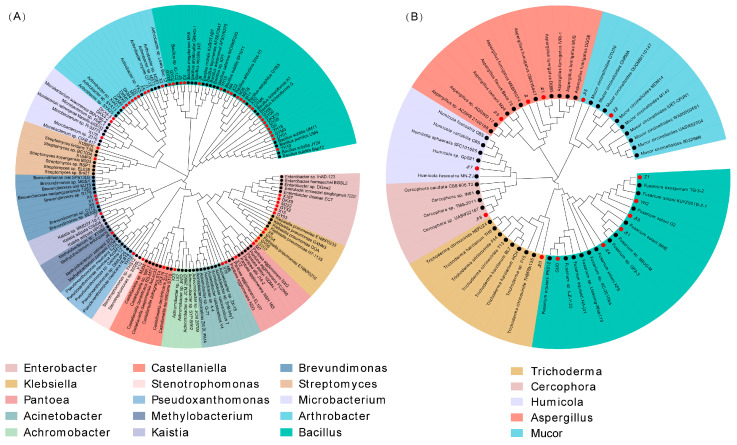
Phylogenetic tree of the 59 isolated bacterial (**A**) and 14 fungal (**B**) strains from the soil on the surface of crop straw. Different colors represent different phyla.

**Figure 5:**
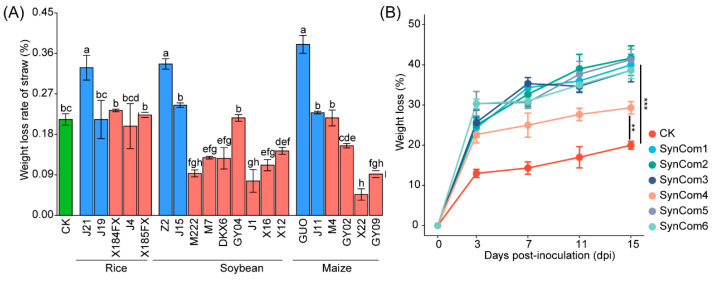
Determination of straw weight loss rate by different strains (**A**) and six SymComs for degradation (**B**). The different-colored columns represent strains from bacteria and fungi, respectively. CK represents sterilized water. Error bars represent standard errors (*n* = 3). Different lowercase letters in the figure indicate significant differences based on Tukey’s test (** *p* < 0.01, *** *p* < 0.001, *p* < 0.05).

To identify microbial strains capable of degrading lignocellulose, we isolated and cultured microorganisms from the soil adhered to the surface of the crop straw. We screened a total of 59 strains of straw-degrading bacteria, which belonged to 14 different families and 19 different genera. Additionally, 13 fungi from six different families and six different genera were screened through the straw medium (Figure 4A,B). Using LEfSe analysis of metagenomic data, we identified 20 cultured strains with higher LDA values from twelve bacterial species and six fungal species. These selected strains were further investigated for their cellulase, xylanase, and laccase production capacity (Supplementary Figure S2, Table S1). We observed that fungi generally exhibited better enzyme-producing abilities compared to bacteria. Notably, *Aspergillus*_sp. J19, *Fusarium*_sp. J11, and *Fusarium_longipes* GUO showed a high capacity to produce cellulase (endo-β-1,4-glucanase) and xylanase (hemicellulase) as well as a significant weight loss rate in the crop straw (Supplementary Figure S4). Finally, we utilized the constructed SynCom system to inoculate the three crop straw species. SynCom2 demonstrated a significantly higher reduction in straw weight loss at 15 DPI (days post inoculation) compared to other SynCom microorganisms (Figure 5, Table S2).

## 4. Discussion

Soil microorganisms play a crucial role in the degradation of organic matter [32]. Crop straw has a high-quality cellulose structural body and serves as a premium carbon source for the soil carbon cycle [4]. Moreover, the abundant microbial communities in the soil collaborate to utilize lignocellulose in the straw as a carbon source for normal functioning [33]. In this study, we observed that the degradation time of three different crop straws significantly altered the bacterial and fungal community diversity and structure. Consistent with prior research, Proteobacteria was identified as the dominant phylum throughout the 180-day degradation experiment [34,35]. Bacteroides were also found to play a crucial role in the breakdown of hemicellulose and xylan, confirming earlier studies [36]. Additionally, fungi play a vital role in straw degradation. For example, *Neurospora crassa* secretes cellulase and xylanase [37], while *Phanerochaete chrysosporium* secretes cellobiose hydrolase and xylanase [38].

Microbial diversity plays a crucial role in system function [39,40]. This study identified the bacterial and fungal strains that played a dominant role in straw decomposition and revealed a strong association with carbon and nitrogen metabolism (Supplementary Figure S1). Additionally, the degradation of straw involves the coordinated action of various enzymes related to lignocellulosic breakdown, such as endo-β-1,4-glucanase, β-glucosidase, xylanase, and laccase [41]. In our investigation, the abundance of genes linked to lignocellulose degradation (e.g., *bglX*, *gmuG*, and *lacZ*) significantly increased as crop straw degradation progressed. This phenomenon is believed to be influenced by the higher carbon-to-nitrogen ratio observed in rice and maize straw compared to soybean straw. Notably, Actinobacteria, Proteobacteria, Chloroflexi, Acidobacteria, and Firmicutes were found to significantly contribute to each CAZyme family, playing a vital role in straw degradation [42,43,44]. These enzymes, crucial for straw degradation, are produced through microbial metabolic activities. This finding aligns with previous research suggesting that microorganisms utilizing lignocellulose produce a substantial number of enzymes associated with straw degradation [45,46,47,48].

Differences among treatment groups and different periods were determined using LEfSe analysis [49]. At the bacterial level, Streptomycetaceae, a crucial family of Actinobacteriota, has been demonstrated to play a significant ecological role in straw degradation [50] and their abundance remains stable during the degradation of plant residues [44,51]. Streptomycetaceae and Bacillaceae can degrade cellulose [52], and *Klebsiella*, a genus of Enterobacteriaceae, also contributes significantly to the cellulose degradation process [53]. Some related bacterial strains were isolated. Fungi also play a pivotal role in the straw degradation process [54]. This study revealed that Ascomycetes and Basidiomycota are key contributors to the straw degradation process [55], with Basidiomycota being the dominant phylum. Measurement of the enzyme production of each strain indicated that the enzyme production capacity of fungi surpassed that of bacteria. For instance, *Aspergillus* and *Fusarium* demonstrated production of excellent lignocellulose degradation-related enzymes [56]. In summary, straws from different crop species recruit distinct degrading bacteria, all of which contribute to straw degradation through enzyme production (Supplementary Figure S4). The SynComs constructed in this study also provide a reference for straw degradation in black soil regions.

## 5. Conclusions

Our study investigated the taxonomy and functional attributes of microbes in soil adhered to the surface of the straw of various crop species during the degradation process, elucidating distinct taxon- and function-specific recruitment strategies. We successfully isolated lignocellulose-degrading bacteria and fungi and identified dominant bacteria and fungi taxa, providing valuable information for future investigations into crop straw degradation. These strains demonstrated an ability to accelerate straw degradation rates, showcasing significant potential applications in crop production systems.

## Data Availability

Data are contained within the article and Supplementary Materials.

## Supplementary Materials

The following supporting information can be downloaded at: https://www.mdpi.com/article/10.3390/microorganisms12050938/s1. Supplementary Figure S1 Linear discriminant analysis effect size (LEfSe) results after 0 and 120 days of straw degradation. Supplementary Figure S2 Functional predictions at the bacteria level using FAPROTAX after 0, 90, and 180 days of straw degradation. Supplementary Figure S3 Comparison of KEGG pathways between 0 and 120 days of crop straw decomposition. Supplementary Figure S4 Determination of enzyme production capacity by different strains. (A) Amount of cellobiohydrolase produced by the strains. (B) Amount of hemicellulase produced by the strains. Supplementary Table S1 Detailed information on strains at the bacterial and fungal levels. Supplementary Table S2 Strains comprising six synthetic microbial communities (Syncoms).

## Appendix A

The raw sequencing reads of amplicon and metagenomic sequencing were deposited into the NCBI Sequence Read Archive Database with the accession numbers of PRJNA919011 and PRJNA918990, respectively.
