# Supplementary material for: Straw from Different Crop Species Recruits Different Communities of Lignocellulose-Degrading Microorganisms in Black Soil
Source: Microorganisms. 2024 May 5;12(5):938. doi: 10.3390/microorganisms12050938 (PMC11123855; doi:10.3390/microorganisms12050938)
Supplement: Supplementary file 1 [file microorganisms-12-00938-s001.zip › microorganisms-2966006-supplementary.pdf]

Supplementary Figures and Tables

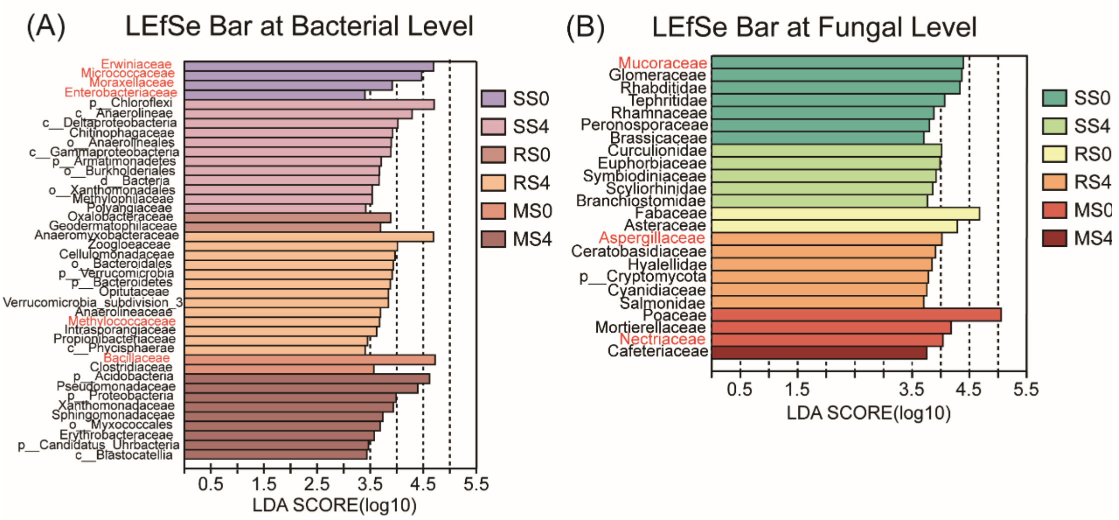

Figure S1 Linear discriminant analysis effect size (LEfSe) results after 0 and 120 days of straw degradation. (A) LEfSe bar plot at the family level of bacteria (LDA value > 3.4). (B) LEfSe bar plot at the family level of fungi (LDA value > 3.7). SS0, RS0, and MS0 represents degradation time of 0 days for the respective straws, while SS4, RS4 and MS4 represents degradation time of 120 days.

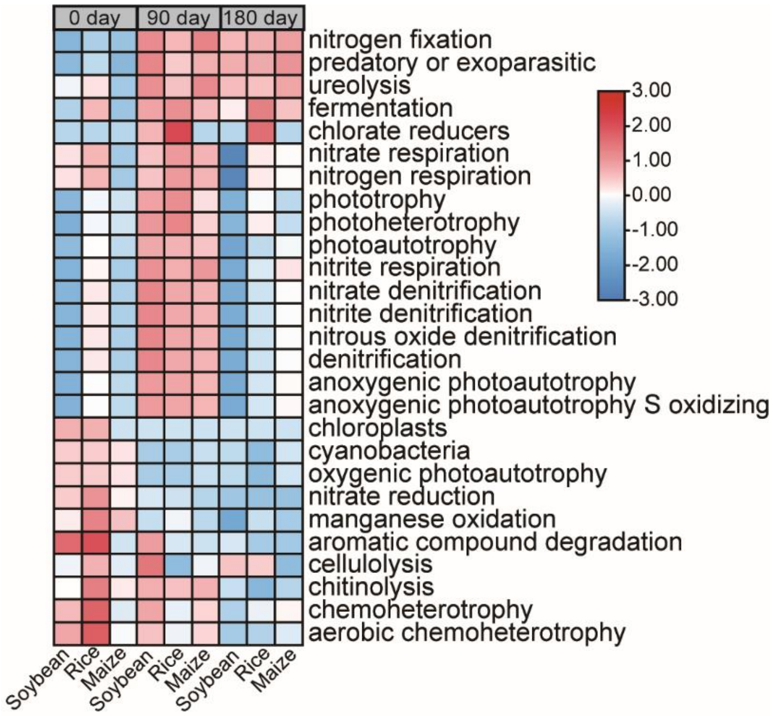

Figure S2 Functional predictions at the bacteria level using FAPROTAX after 0, 90, and 180 days of straw degradation.

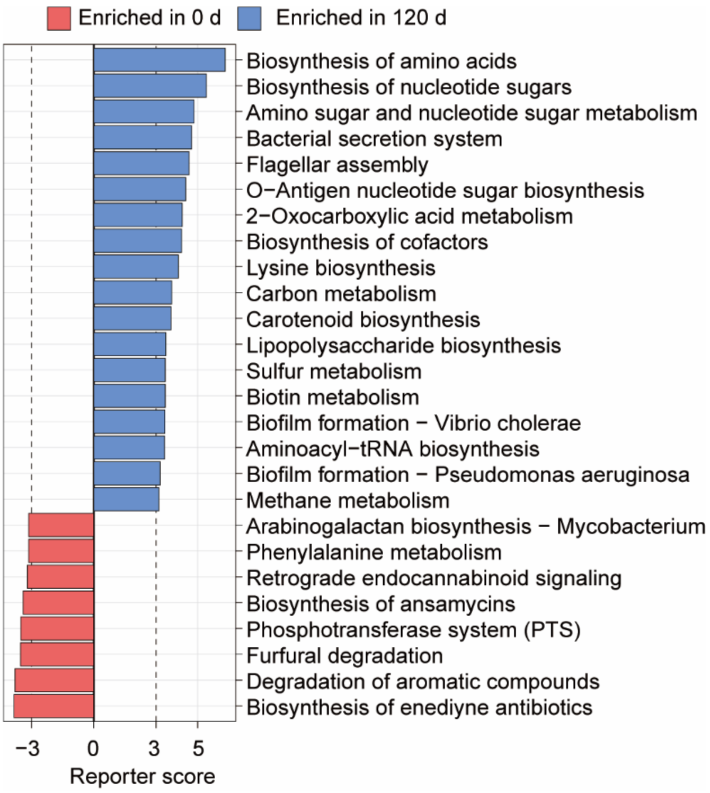

Figure S3 Comparison of KEGG pathways between 0 and 120 days of crop straw decomposition. The plot displays the reporter score versus $-\log_{10}P$ value from the hypergeometric test. Vertical dashed lines indicate a reporter score of ±3.

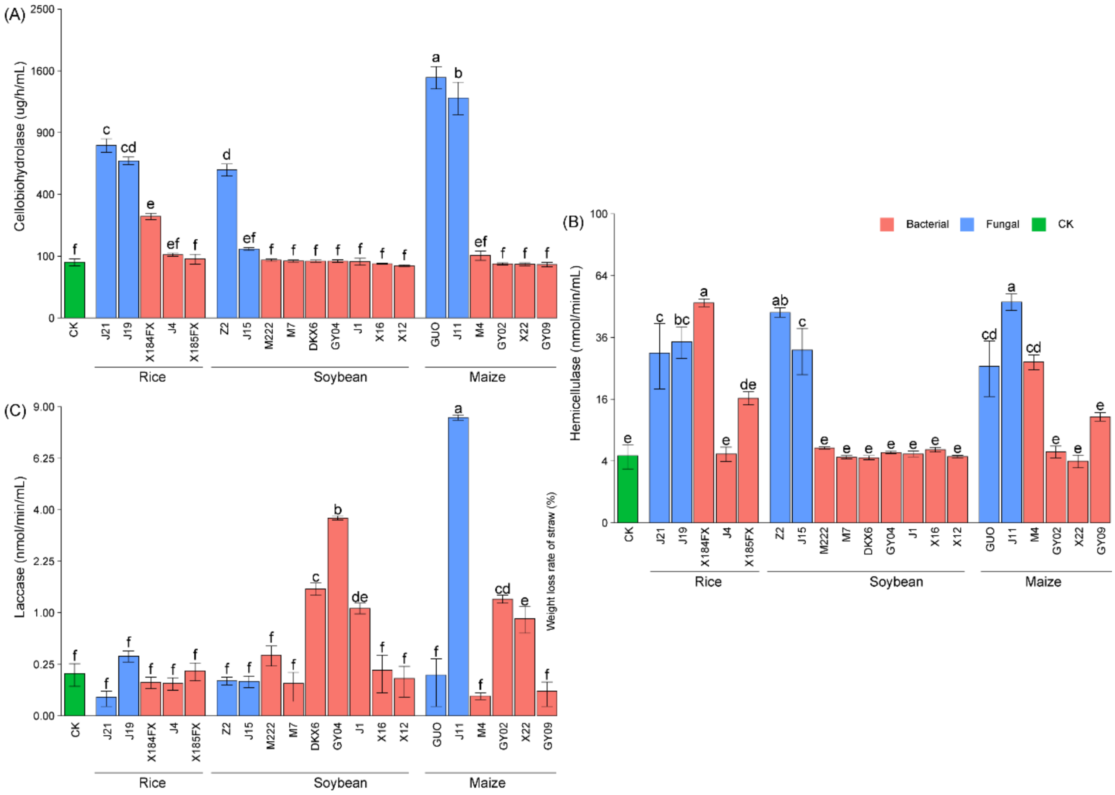

Figure S4 Determination of enzyme production capacity by different strains. (A) Amount of cellobiohydrolase produced by the strains. (B) Amount of hemicellulase produced by the strains. (C) Amount of laccase produced by the strains. Red indicates fungi, and black indicates bacteria. The different-colored columns represent strains from bacteria and fungi. CK represents sterilized water. Error bars represent standard errors (n = 3). Different lowercase letters in the figure indicate significant differences based on Tukey's test ($p < 0.05$).

Table S1 Detailed information on strains at the bacterial and fungal levels.

Taxonomy characteristics	Strain name	Species information	Source of strain
Bacterial	DKX6	*Glutamicibacter uratoxydans*	Soybean straw
	GY02	*Priestia megaterium*	Maize straw
	GY04	*Klebsiella pneumoniae*	Soybean straw
	GY09	*Bacillus velezensis*	Maize straw
	M4	*Bacillus altitudinis*	Maize straw
	M7	*Pantoea agglomerans*	Soybean straw
	M222	*Enterobacter hormaechei*	Soybean and Rice straw
	X12	*Pantoea vagans*	Soybean straw
	X16	*Acinetobacter NGCT_s*	Soybean straw
	X22	*Bacillus altitudinis*	Maize straw
	X184FX	*Streptomyces daghestanicus*	Rice straw
	X185FX	*Streptomyces albidoflavus*	Rice straw
Fungal	GUO	*Fusarium longipes*	Maize straw
	J11	*Fusarium sp.*	Maize straw
	J15	*Mucor circinelloides*	Soybean straw
	J19	*Aspergillus sp.*	Rice straw
	J21	*Aspergillus fumigatus*	Rice straw
	Z-2	*Mucor circinelloides*	Soybean straw

Table S2 Strains comprising six synthetic microbial communities (Syncoms).

Synthetic microbial communities (SynComs)	Species information
SynCom1	*Aspergillus* sp. J19
	Fusarium sp. J11
	Fusarium longipes GUO
	Mucor circinelloides J15
SynCom2	*Fusarium* sp. J11
	Streptomyces daghestanicus X184FX
	Mucor circinelloides Z-2
	Aspergillus sp. J19
SynCom3	*Fusarium longipes* GUO
	Fusarium sp. J11
	Aspergillus fumigatus J21
	Aspergillus sp. J19
SynCom4	*Fusarium* sp. J11
	Klebsiella pneumoniae GY04
	Glutamicibacter uratoxydans DKX6
	Priestia megaterium GY02
SynCom5	*Fusarium longipes* GUO
	Mucor circinelloides Z-2
	Aspergillus fumigatus J21
	Mucor circinelloides J15
SynCom6	*Aspergillus* sp. J19
	Fusarium sp. J11
	Fusarium longipes GUO
